# The inverse effect of meal intake on controlled attenuation parameter and liver stiffness as assessed by transient elastography

**DOI:** 10.1186/s12876-017-0609-6

**Published:** 2017-04-13

**Authors:** Kanittha Ratchatasettakul, Sasivimol Rattanasiri, Kwannapa Promson, Pranee Sringam, Abhasnee Sobhonslidsuk

**Affiliations:** 1 Division of Gastroenterology and Hepatology, Department of Medicine, Faculty of Medicine, Ramathibodi Hospital, Mahidol University, 270 Rama 6 Road, Bangkok, 10400 Thailand; 2 Section for Clinical Epidemiology and Biostatistics, Faculty of Medicine, Ramathibodi Hospital, Mahidol University, Bangkok, 10400 Thailand

**Keywords:** Controlled attenuation parameter, Liver stiffness, Transient elastography, Meal, Steatosis, Fibrosis

## Abstract

**Background:**

Controlled attenuation parameter (CAP) and liver stiffness (LS) measured by transient elastography (TE, Fibroscan®) have been used for steatosis and fibrosis assessment. We evaluated the effect of meal intake on CAP and LS values.

**Methods:**

Forty patients who had had a liver biopsy within the previous month were recruited. The biopsy was graded for fibrosis (F) and steatosis (S) stagings. TE was performed after overnight fasting (baseline values) and 15, 30, 45, 60, 90, and 120 min following the intake of a standard commercial formula meal, and every 30 min until LS and CAP values returned to baseline. The effect of meal intake on CAP and LS values was analyzed with a multilevel mixed model approach*.*

**Results:**

The mean age was 53.1 ± 11.2 years old. The mean (SD) BMI was 25.6 ± 4.5 kg/m^2^. F0, F1, F2, F3 and F4 fibrosis stages were found in 17 (42.5%), 9 (22.5%), 4 (10.0%), 8 (20.0%) and 2 (5.0%), respectively. S0, S1, S2 and S3 steatosis stages were seen in 22 (55.0%), 11 (27.5%), 4 (10.0%) and 3 (7.5%), respectively. The mean (SD) CAP and median (IQR) LS values at baseline were 249.7 ± 58.1 dB/m and 11.9 (6–18.1) kPa. A significant decrease in CAP values was observed in all patients 15 to 120 min after meals, with the CAP peak value at 60 min and the mean post-meal delta reduction of 18.1 dB/min. CAP values declined after meals at early fibrosis stages and across all stages of steatosis. A significant increase in LS values after meal intake was observed within 15 to 120 min, with the LS peak value at 15 min and the mean post-meal delta increase of 2.4 kPa. Post-meal CAP and LS values returned to baseline within 150 min following meals.

**Conclusion:**

Following a meal, patients’ CAP values declined with the peak value at 60 min, contrasting with the rising of LS values with the peak value at 15 min. The post-meal CAP and LS values returned to baseline by 150 min. A fasting period of more than 150 min after a meal is recommended for patients undergoing TE.

## Background

Hepatic steatosis is a common feature in many types of chronic liver disease such as alcoholic liver disease, nonalcoholic fatty liver disease (NAFLD), and chronic viral hepatitis B and C [1, 2]. NAFLD is a frequent concomitant condition in chronic liver disease that can accelerate the progression of the disease [3]. NAFLD can reduce virological response in treatment of chronic hepatitis C [4]. Liver biopsy is a gold standard for the assessment of steatosis and other histological features [5]. However, this procedure is invasive and costly. Moreover, sampling errors and questionable reproducibility in liver biopsy have been reported [5–7].

A novel, non-invasive tool based on ultrasound attenuation, the controlled attenuation parameter (CAP), was developed to assess the degree of liver steatosis [8]. It is performed with transient elastrography (TE, FibroScan®). The reliable diagnostic performance of CAP has been confirmed in many types of chronic liver disease, including chronic viral hepatitis and NAFLD [9, 10]. Previous studies demonstrated that liver stiffness (LS), as assessed by TE, was affected by food consumption from the mechanism of postprandial hyperemia [11–13]. High body mass index (BMI), alcohol drinking, histological steatosis grade 3 and CAP > 323 dB/m interfere with the accuracy of CAP measurement [14]. The effect of a meal on CAP has never been investigated. We aimed to evaluate the effect of a meal on CAP and LS. We hypothesized that CAP value might increase after food ingestion similar to the phenomenon of the meal effect on LS.

## Methods

### Patients

Patients with chronic liver diseases who underwent a percutaneous liver biopsy at the liver clinics of Ramathibodi Hospital between January 2015 and January 2016 were recruited. A liver biopsy was performed to identify the cause of abnormal liver tests or for staging of chronic liver diseases. Inclusion criteria were: (1) age > 18 years old (2) interpretable liver biopsies including the length of the liver > 10 mm. Exclusion criteria were: (1) contraindicated to TE (e.g. having congestive heart failure, pregnancy, ascites, hepatitis and/or cholestasis jaundice (aspartate aminotransferase (AST) or alanine aminotransferase (ALT) > five times upper normal, total bilirubin > 5 mg/dl), (2) TE failure (interquartile range/median was more than 0.3, a success rate lower than 60%), (3) regular alcoholic drinking (>20 g/day), (4) end-stage renal disease, (5) refusal to participate with the study. The study was conducted according to the principles of the Declaration of Helsinki (revision of Edinburgh, 2000). The study was reviewed and approved by the Committee on Human Right Related to Research Involving Human Subjects, Faculty of Medicine, Ramathibodi Hospital, Mahidol University (ID 11-57-22). Written informed consent was obtained before the beginning of the study.

### Clinical evaluation and biochemical data

Patients underwent abdominal ultrasonography and anthropometric measurement including body mass index (BMI) (weight (kg)/height (m^2^)), waist circumference and hip circumference. Demographic data were obtained from medical records. Biochemical data including ALT, alkaline phosphatase (ALP), albumin, total bilirubin, fasting blood sugar (FBS), triglyceride (TG), high-density lipoprotein cholesterol (HDL), serum creatinine, were collected within 4 weeks of the liver biopsy.

### Liver biopsy

Patients underwent ultrasound-guided percutaneous liver biopsy. Liver biopsy was performed within 4 weeks of the study recruitment. Liver pathology was evaluated by a single experienced hepatopathologist who did not have access to clinical data of the study patients. Liver histology was graded for fibrosis (F) and steatosis (S) staging using Metavir scoring (F0-F4) and steatosis staging (: S0, < 5%, S1, 5–33%; S2, 34–66%; and S3, > 66%) [2, 15].

### Ultrasonography

Patients underwent abdominal ultrasonography (HITACHI, Japan) using a convex 3.5 MHz probe for evaluation of fatty liver on the same day with TE. Fatty liver was evaluated based on the degree of liver brightness, the presence of sonographic contrast appearance between liver and kidney parenchyma, reduced vessel wall echogenicity, high posterior attenuation and poorly delineated diaphragm [16].

### Measurement of liver stiffness (LS) and controlled attenuation parameter (CAP)

A single operator who had experiences of more than 1000 cases of TE measurement performed TE in this study. While the patients were lying on their back, the tip of a 3.5 MHz standard M probe was placed on the skin of intercostal space over the right lobe of the liver. The liver stiffness was measured with transient elastography (FibroScan®, Echosens, Paris, France). LS is expressed in kilopascal (kPa). The only results accepted followed the standard protocol with 10 valid measurements and had success rates over 60% and an interquartile range (IQR)/median of less than 0.3 [17].

CAP is a novel non-invasive parameter to assess the degree of steatosis by using a vibration-controlled transient elastography (VCTE™) device based on the properties of ultrasonic signals acquired by TE. A total ultrasonic attenuation (go-and-return path) was estimated. CAP was evaluated using the same radiofrequency data and region of interest used for the assessment of LS. CAP values were expressed in dB/m, range 100–400 dB/m [8]. CAP value was interpreted only when the associated LS value was valid.

LS and CAP measurement were obtained simultaneously after an eight-hour overnight fasting (baseline values) and 15, 30, 45, 60, 90, and 120 min following the intake of a standard commercial formula (400 ml, 600 kcal, protein: carbohydrates: fat - 15: 55: 30 (equivalent to a standard Thai meal)), and every 30 min until the LS and CAP values returned to baseline.

### Statistical analysis

For descriptive analysis, continuous variables were expressed as mean ± standard deviation (SD) or median (Interquartile range - IQR), and categorical variables as number (%). The multilevel mixed model approach was used to analyze the effect of a meal on CAP and LS at each time point compared to the baseline (or premeal values). We controlled for BMI and time by including both variables in the mixed linear models of CAP and LS. *P* < 0.05 was taken as statistical significance. Statistical analysis was performed with Stata software version 14.

## Results

### Characteristics of patients

Seventy-five patients underwent liver biopsy during the study period. Thirty-five were excluded due to the following reasons: end-stage renal disease (11), markedly elevated ALT or bilirubin (5), TE failure (2) or refusal to participate in the study (17). A total of 40 patients were enrolled to the study. Baseline characteristics and biochemical data are shown in Table 1. The majority of study patients (28/40, 70%) were female. The mean age was 53.1 ± 11.2 years old. Chronic hepatitis B was the most common cause of liver disease (12/40, 30%). Regarding the staging of liver fibrosis, there were 17 patients (42.5%), 9 (22.5%), 4 (10.0%), 8 (20.0%), and 2 (5.0%) with F0, F1, F2, F3, and F4 stages, respectively (Table 2). According to steatosis staging, 22 (55.0%), 11 (27.5%), 4 (10.0%) and 3 (7.5%) of the patients had S0, S1, S2 and S3 stages, respectively. The mean (SD) CAP and median (range) LS at baseline were 249.7 ± 58.1 dB/m and 11.9 (6–18.1) kPa.

**Table 1:** Demographic and laboratory data

Characteristics	Patients (*n* = 40)
Female^a^	28 (70.0%)
Age, year ^b^	53.1 ± 11.2
BMI, kg/m^2b^	25.6 ± 4.5
Waist circumference, cm^b^	88.4 ± 11.9
Hip circumference, cm^b^	97.9 ± 8.1
Chronic Hepatitis B^a^	12 (30.0)
Chronic Hepatitis C^a^	5 (12.5)
NAFLD^a^	8 (20)
Other^a^	15 (37.5)
Biochemical parameters	
AST, U/L^b^	71.9 ± 35.6
ALT, U/L^c^	92.5 (59.5–153.5)
FBS, mg/dl^b^	113.2 ± 41.4
Triglyceride, mg/dl^b^	117.7 ± 65.5
HDL, mg/dl^b^	50.4 ± 18.3

*SD* standard deviation, *NAFLD* nonalcoholic fatty liver disease, *BMI* body mass index, *AST* aspartate aminotransferase, *ALT* alanine aminotransferase, *ALP* alkaline phosphatase, *FBS* fasting blood glucose, *HDL* high density lipoprotein

^a^number (%)

^b^mean ± SD

^c^median (IQR)

**Table 2:** Radiological and histological data

Characteristics	Patients (*n* = 40)
Liver biopsy	
Fibrosis stage	
F0/F1/F2/F3/F4^a^	17 (42.5)/9 (22.5)/4 (10.0)/8 (20.0)/2 (5)
Steatosis stage	
S0/S1/S2/S3^a^	22 (55.0)/11 (27.5)/4 (10.0)/3 (7.5)
Transient elastography (Premeal)	
CAP, dB/m^b^	249.7 ± 58.1
LS, kPa^c^	11.9 (6–18.1)
Ultrasonography	
No fatty liver^a^	17 (46)
Mild fatty liver^a^	14 (37.8)
Moderate fatty liver^a^	6 (16.2)

*Abbreviations*: *SD* standard deviation, *NAFLD* nonalcoholic fatty liver disease, *BMI* body mass index, *AST* aspartate aminotransferase, *ALT* alanine aminotransferase, *ALP* alkaline phosphatase, *FBS* fasting blood glucose, *HDL* high density lipoprotein, *CAP* controlled attenuation parameter, *LSM* liver stiffness

^a^number (%)

^b^mean ± SD

^c^median (IQR)

### The effect of a meal on CAP and LS

**Fig. 1:**
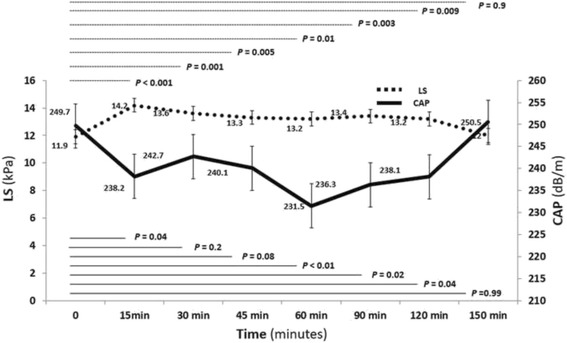
Controlled attenuation parameter (CAP) and liver stiffness (LS) after meal intake

CAP and LS values were assessed after overnight fasting (baseline values) and at 15, 30, 45, 60, 90, 120 min following the intake of a standard commercial formula, and every 30 min until CAP and LS values returned to baseline. A significant decline of CAP values following meal intake was observed 15 to 120 min (*P* < 0.05) in Fig. 1. The CAP post-meal peak value was seen at 60 min (*P <* 0.01)*.* All post-meal CAP values returned to baseline by 150 min. The peak post-meal delta decrease of CAP was 18.1 dB/m (*P* < 0.01)*.* A significant increase in LS values following meal intake was observed 15 to 120 min (*P* < 0.01), with the LS post-meal peak value at 15 min (*P* < 0.001)*.* Post-meal LS values returned to baseline by 150 min in all. The peak post-meal delta increase of LS was 2.4 kPa (*P* = 0.001).

### The effect of meal on CAP according to fibrosis and steatosis stages

**Fig. 2:**
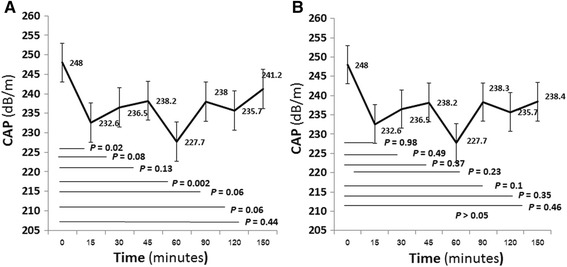
Controlled attenuation parameter (CAP) after meal intake stratified by Metavir stage of liver fibrosis. **a** F0-F2. **b** F3-F4

For thirty (75%) patients with F0-F2 stage, the mean CAP values following meal intake began to decline from 15 min (*P* = 0.02) and peaked at 60 min (*P* = 0.002) (Fig. 2a). The peak post-meal delta decrease of CAP values was 20.3 dB/m (*P =* 0.002). The CAP values returned to baseline by 90 min. There were 10 (25%) patients who had F3-F4 fibrosis stage (Fig. 2b). The mean CAP values following meal intake did not show significant change.

**Fig. 3:**
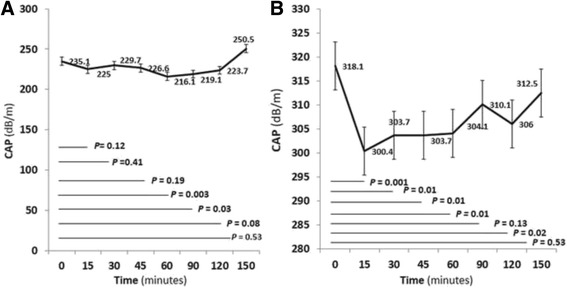
Controlled attenuation parameter (CAP) after meal intake stratified by steatosis stage. **a** S0-S1. **b** S2-S3

Thirty-three (82.5%) patients had S0-S1 steatosis stage. The mean CAP values following meal intake was significant decline at 60 min (*P* = 0.003) and at 90 min (*P* = 0.03), respectively (Fig. 3a). The mean post-meal CAP peak value was at 60 min. The mean CAP values returned to baseline by 120 min. There were 7 (17.5%) patients who had S2-S3 steatosis stage. The mean CAP values following meal intake declined 15 to 120 min (*P* < 0.05) (Fig. 3b). The mean post-meal CAP peak value was at 15 min and the CAP values returned to baseline by 150 min.

### The effect of meal on LS according to fibrosis and steatosis stages

**Fig. 4:**
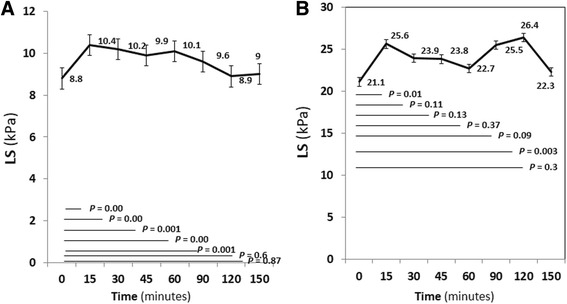
Liver stiffness (LS) after meal intake stratified by Metavir stage of liver fibrosis. **a** F0-F2. **b** F3-F4

Thirty (75%) patients had F0-2 fibrosis stage, the mean LS values following meal intake was significantly increase at 15 min to 120 min (Fig. 4a). The peak of increment was at 15 min. The mean LS values returned to baseline at 150 min. There were 10 (25%) patients who had F3-4 fibrosis stage. The mean LS values following meal intake showed significantly increase at 15 and 120 min (*P* = 0.01 and *P* = 0.003) (Fig. 4b). The peak of increment was at 120 min. The mean LS values returned to baseline at 150 min.

**Fig. 5:**
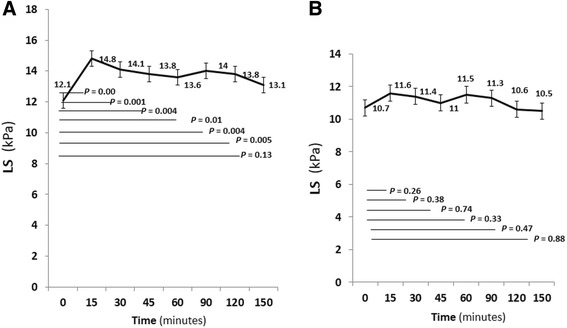
Liver stiffness (LS) after meal intake stratified by steatosis stage. **a** S0-S1. **b** S2-S3

Thirty-three (82.5%) patients had S0-S1 steatosis stage. The mean LS values following meal intake showed significantly increase at 15 to 120 min (Fig. 5a). The peak of increment was at 15 min. The mean LS values returned to baseline at 150 min. There were 7 (17.5%) patients who had S2-S3 steatosis stage. The mean LS values following meal intake did not show any change over the time (Fig. 5b).

## Discussion

We recruited 40 patients who had liver biopsy to the study. Contrary to our primary assumption, the CAP values were significantly reduced after meal intake 15 min to 120 min, and the values returned to the premeal level by 150 min. The CAP post-meal peak was observed at 60 min after meal intake. We found the same effect of meal intake on CAP values at early stages of fibrosis and across all stages of steatosis. The effect of meal on CAP values in the opposite direction with the effect of meal on LS values was showed in our study. The rising of LS values after meal in this study was similar to previous reports [11–13, 18]. Undergoing transient elastography without adequate fasting can lead to falsely decreased CAP value of −18.1 dB/m and incorrectly increased LS value of +2.4 kPa in average. LS is calculated from velocity. Velocity is related to tissue stiffness, the harder the tissue, the faster the shear wave propagation. However, for CAP, it measures the ultrasound attenuation. So, post-prandial hyperemia, LS increased while CAP decreased from reducing attenuation [19]. Due to the fact that 70% of study patients were female and 30% of liver disease were chronic hepatitis B and the mean BMI of subjects was 25.6 ± 4.5 kg/m^2^, the generalizability of the results study should be cautious. The accuracy of CAP measurement for steatosis determination is low in the patients with obesity and advanced fibrosis [20]. Furthermore, we did not have a control group with normal water taking to exclude the influence of liquid volume as in the study of Mederacke I, et al. [11].

The LS values were significantly elevated after meal intake 15 to 120 min, and they returned to baseline by 150 min. The LS post-meal peak value was observed at 15 min. However, the changing of LS values after meal in our study had somewhat differences from previous reports in term of the returning time to baseline level [11, 12]. An elevation of postprandial LS value was observed 15 to 45 min after meal intake, with returning to baseline level within 120 min in that study [12]. Because only patients with chronic hepatitis C were included [12], the difference in the etiology of liver diseases may explain some dissimilar findings. Furthermore, from the study by Mederacke et al., a standardized continental breakfast, which consisted of two rolls, ham, cheese, butter and jam with approximately 600 kcal, 54% carbohydrates, 26% fat and 20% protein, an elevation of LS values followed meal intake occurred 15 to 60 min, and returned to baseline at 180 min [11]. Although the meal in our study and that of Mederacke et al. was similar in the amount of energy and the proportion of nutrients, the returning time of LSM values to baseline in their study was 30 min longer. This phenomenon may be explained by the different types of diet in the two studies. Liquid meal was chosen in our study instead of solid meal. Moreover, LS values at 150 min were not performed in that study [11]. To the best of our knowledge, our report is the first experimental study to explore the effect of meal on CAP in addition to LS values.

There is some limitation in our study. The protocol was not designed to explain the mechanism that resulted in the changing of CAP and LS values following meal intake. Furthermore, it was conducted with a small number of patients and as a single center study. This topic requires further research in a larger population to confirm our findings.

## Conclusions

CAP values declined after meal intake with the post-meal peak value at 60 min, contrasting with the rising of LS values after meal intake with the post-meal peak value at 15 min. TE performed without adequate fasting can yield a falsely lower value of CAP and misleadingly higher value of LS. A fasting period of more than 150 min after a meal is recommended for patients undergoing TE for measuring LS and CAP.

## Abbreviations

ALP: Alkaline phosphatase
ALT: Alanine aminotransferase
AST: Aspartate aminotransferase
BMI: Body mass index
CAP: Controlled attenuation parameter
F: Fibrosis
FBS: Fasting blood sugar
HDL: High density lipoprotein cholesterol
IQR: Interquartile range
LS: Liver stiffness
NAFLD: Nonalcoholic fatty liver disease
S: Steatosis
TE: Transient elastography
TG: Triglyceride
